# Potential Mechanisms underlying the Protective Effect of Pregnancy against Breast Cancer: A Focus on the IGF Pathway

**DOI:** 10.3389/fonc.2016.00228

**Published:** 2016-10-26

**Authors:** Tiffany A. Katz

**Affiliations:** ^1^Center for Precision Environmental Health, Baylor College of Medicine, Houston, TX, USA

**Keywords:** insulin-like growth factor I, pregnancy, mammary gland biology, breast cancer risk, breast cancer prevention

## Abstract

A first full-term birth at an early age protects women against breast cancer by reducing lifetime risk by up to 50%. The underlying mechanism resulting in this protective effect remains unclear, but many avenues have been investigated, including lobular differentiation, cell fate, and stromal composition. A single pregnancy at an early age protects women for 30–40 years, and this long-term protection is likely regulated by a relatively stable yet still modifiable method, such as epigenetic reprograming. Long-lasting epigenetic modifications have been shown to be induced by pregnancy and to target the IGF pathway. Understanding how an early first full-term pregnancy protects against breast cancer and the role of epigenetic reprograming of the IGF system may aid in developing new preventative strategies for young healthy women in the future.

## Introduction

An early first full-term birth (FFTB) is the most effective modifiable breast cancer prevention method, with the potential of reducing a woman’s lifetime risk up to 50%. The first documented observation of this preventative behavior was by Bernardino Ramazzini in 1700. He noted that “tumors of the breast are found more often in nuns than any other women” and speculated that this was due to a life of celibacy (1). A landmark case–control study revisited this phenomenon in 1970, finding that compared to nulliparous women, women who underwent their FFTB before the age of 20 had a risk reduction of 50%. In this study of 17,022 women (4323 cases and 12,699 controls) in 7 regions around the world, a strong positive linear relationship between age at FFTB and breast cancer risk was observed (2). Since then, others have reproduced these findings showing that women who experience their FFTB before the age of 20 or 25 reduce their lifetime risk by 50 or 38%, respectively (3–5). An increased number of births is also associated with decreased breast cancer risk, and these associations are stronger for estrogen receptor (ER)+ disease (6–10).

The abovementioned study identified a positive linear correlation between age at FFTB and breast cancer risk (2). Nulliparous women were used as the reference group. Women who give birth for the first time above the age of 33 were no longer protected against breast cancer compared to nulliparous women. In fact, those women were now at an increased risk compared to nulliparous women. This is especially concerning since the average age at FFTB in the United States has been increasing over time (11). The proportion of women who gave birth for the first time between the ages of 30 and 34 rose 28% (from 16.5 to 21.1%) and those over 35 years of age rose 23% (from 7.4 to 9.1%) between 2000 and 2014 (11). Learning more about the mechanism underlying the protective effect of an early FFTB against breast cancer could help to provide protection for women giving birth at an older age.

The protective effect of pregnancy has been replicated in multiple animal models including mice and rats using both carcinogen and spontaneous carcinoma models (4, 12, 13). Simply replicating the hormonal milieu of pregnancy has been shown to induce robust protection against mammary tumors (14–16). Given at a dose resulting in circulating levels similar to pregnancy, estradiol (E2) alone decreases the percent of tumor bearing animals in response to a carcinogen; and E2 in combination with progesterone (P4) enhances this protective effect presumably by creating an environment even more similar to a pregnant state (14, 15). Additionally, the parous mammary gland is less successful in supporting the development of hyperplastic lesions or tumors. In a syngeneic rat model, carcinogen-treated epithelial cells were transplanted into uniparous, age-matched virgin (AMV), or young virgin (YV) rats. Compared to either group of virgin rats, transplants into uniparous rats had fewer hyperplasias and adenocarcinomas (17).

The stage of the pregnancy cycle (i.e., pregnancy, lactation, or involution) most important in reducing breast cancer risk is unclear. The previously mentioned study (2) chose to include regions across the globe in order to capture women who breastfed for extended periods of time and women who rarely breast fed but was unable to confirm a link between breast feeding and breast cancer risk. Other studies have reported associations between breast feeding and breast cancer risk. In a meta-analysis of 31 studies, 27 included breast feeding data and 13 of those found that longer durations of breast feeding protected against breast cancer (18). The current recommendation by physicians is that breast feeding for longer than 2 years over a woman’s lifetime is protective against breast cancer, and this is supported by multiple studies. Breast feeding for as little as 6 months (19) provides a significant reduction in breast cancer risk, but longer durations of breast feeding are even more beneficial (20–23).

Surprisingly, in a rodent model, lactation is not obligatory to elicit protection against mammary cancer. Parous rats that were not permitted to nurse and animals that did nurse were equally protected from mammary cancer (24). Additionally, the lactogenic drug perphenazine was not sufficient in protecting against mammary cancer (14).

## Potential Mechanisms: Lobular Differentiation, Cell Fate, and Stromal Composition

### Lobular Differentiation

In an attempt to identify the underlying cause of parity-induced protection against breast cancer, several morphogenic and molecular mechanisms have been investigated including differentiation state, cell fate, and stromal alterations of the mammary gland. One of the first comprehensive studies investigating differentiation of the breast reported that parous women displayed a higher proportion of differentiated lobules in the breast. Four lobule types were identified with type 1 being the least and type 4 being the most differentiated. Cells isolated from the least differentiated lobule type were the most susceptible to carcinogenic insults, displaying increased survival efficiency and multinucleation (25). Of peak importance, parous women who developed breast cancer displayed lobular profiles identical to nulliparous women. This indicates that these women may not have undergone the entire differentiation cycle required for the protective effect of parity against breast cancer (26–28).

Interestingly, in a more recent report, proportions of differentiated and undifferentiated lobules were not different between parous and nulliparous women (29). The two studies used distinct lobule identification techniques and sampling methods. The 2014 study used previously established imaging and quantitation criteria, described by Russo (26), and Milanese et al. (30), to determine the proportion of lobule types represented in each specimen. When comparing samples from nulliparous women and women >10 years postpartum (*n* = 10), there was no difference in lobular composition of the breast (29). The more recent study included women who underwent clinically indicated breast biopsies including only one specimen per sample, whereas the previously mentioned studies included whole breast or lumpectomy samples, which were divided several times providing numerous samplings per patient. These differences may have contributed to the discrepancy between studies.

It has been hypothesized that type 1 lobules in parous women may all appear histologically the same, but some may have regressed from a type 3 or 4 lobule and may actually be functionally and molecularly different from immature type 1 lobules in nulliparous women. This lobular regression has been termed age-related lobular involution. Studies investigating age-related lobular involution have found that women with predominately type 1 lobules and no type 3 lobules (i.e., lobular proportions similar to nulliparous women in the first study), have likely undergone lobular involution, and actually have a decreased risk for breast cancer (30, 31). These epidemiological data support the study by Jindal et al.; their finding may indicate that in women >10 years postpartum age-related involution may have occurred.

### Cell Fate

While controversial, it is believed that changes in cell fate may contribute to the protective effect of pregnancy (32). Pregnancy modulates mammary stem cell number and function, but the long-term implications of these changes are unclear (33). Basal stem cells and alveolar progenitor cells populate the gland in preparation for lactation (34, 35). Shifts in these populations of cells could contribute to the protective effect of parity against breast cancer (36), but this is not a consistent finding (12, 37). Pathways involved in cell fate (wnt/notch) have been shown to be significantly altered with parity in both rodent models and women. In a murine gene expression array study, wnt2 was downregulated in the mouse involuted mammary gland compared to nulliparous controls (38). Additionally, the ligand wnt4 and the number of wnt4-secreting cells were dramatically reduced in parous mammary glands compared to nulliparous (39, 40). Of additional importance, wnt4 is lower in breast tissue from parous women who also display a reduction in CD44^+^p27^+^ cells (41). p27 is known to affect the number and proliferation of stem cells and may therefore indicate a reduction in the number of mammary progenitor cells in parous breast tissue.

### Stromal Composition

Dramatic alterations in the parous stromal microenvironment have been well documented. The tissue microenvironment is extremely plastic in the breast especially during pregnancy. At this time, there is a great proliferative effort in the epithelial compartment, while the adipose cells are reduced and acini are formed. Acini begin producing and releasing milk, and the breast becomes engorged for lactation. During involution, the gland undergoes a dramatic reconstruction very similar to wound healing. During these stages, the gland becomes filled with activated fibroblasts and immune infiltration (42). These changes contribute to an enriched extracellular matrix (ECM), which becomes stiffer and more collagen dense with an increased stroma/parenchyma ratio (38, 43–46). Gene expression studies in rodents and humans have supported these findings showing that ECM and immune gene signatures can differentiate between parous and nulliparous breast tissue (47–49).

Of greater interest is the role these stromal alterations play in preventing tumorigenesis. In a syngeneic rat model, the postpartum epithelial structure was required for pseudopregnancy to protect the gland from breast cancer (50). In this study, epithelial cells were isolated from mice treated with a carcinogen and transplanted into parous animals, which either had their fat pad cleared prior to puberty or retained complete epithelial structures. Transplants into animals with a cleared fat pad displayed increased hyperplasia and carcinoma growth, while animals with full epithelial structure, which had been exposed to a pregnancy, were protected from carcinogenesis (50). In a similar study, mammary epithelial cells isolated from animals that were treated with a carcinogen were implanted into YVs, AMVs, and uniparous animals with cleared fat pads. Significantly fewer transplants grew in uniparous animals (14%), compared to 33% in AMV and 55% in YV, and significantly fewer hyperplasias developed in the transplants in uniparous animals (17). These data indicate that both the presence of epithelial cells and the stromal status are important in determining outgrowth of tumorigenic cells.

The ECM composition has been shown to effect breast cancer cell growth (51–56). Particularly, the stiffness of the matrix can influence which signaling cascades become activated, as well as migration and cellular organization phenotypes. In response to prolactin, T47D cells in a high density/stiff matrix migrate less and signal through the FAK/ERK pathway, while in low density/compliant matrix, they will migrate more but signal through the canonical JAK/STAT pathway (52). In a follow-up report, the interaction between E2 and prolactin was highly influenced by matrix stiffness (51). Since stromal composition and density are dramatically effected by parity, it is likely that these factors work together to influence the protective effect of parity against breast cancer.

## A Role for Insulin-Like Growth Factor I in the Protective Effect of Pregnancy: Epidemiological Data and Rodent Models

### Epidemiological Data

Insulin-like growth factor I (IGFI) in circulation has been shown in many studies to be associated with breast cancer risk (57–59). In a nested case–control study using the Nurses Health Study data, in premenopausal women, higher circulating IGFI was associated with a significant increase in breast cancer risk (60). Increased breast cancer risk was also associated with higher circulating IGFI measured during the first pregnancy (61). The Northern Sweden Maternity Cohort used a nested case–control design including 244 cases and 453 controls. In this study, circulating IGFI was higher in cases than controls and breast cancer risk increased significantly with higher IGFI tertiles. Compared to the lowest tertile, women in the highest IGFI tertile had a 73% increase in breast cancer risk (61). However, this was not replicated in two other studies: one using the AVON Longitudinal Study of Parents and Children Cohort (62) and another in the Finnish Maternity Cohort (63). Therefore, chronic exposure to high circulating IGFI may have a bigger impact than exposure to elevated levels during pregnancy.

While it is well accepted that circulating IGFI levels are associated with breast cancer risk, interestingly, parity is associated with lower circulating IGFI levels. In the Nurses Health Study, 1037 healthy women were used to investigate circulating IGFI and lifestyle factors. This study identified age, smoking, parity, and hormone use to be significantly associated with circulating IGFI levels (64). Circulating IGFI was significantly lower in parous women and was inversely correlated with number of births, which parallels the data on breast cancer risk and parity (64). These observations support the hypothesis that reduced IGFI postpartum contributes to the protective effect of pregnancy against breast cancer.

**Figure 1:**
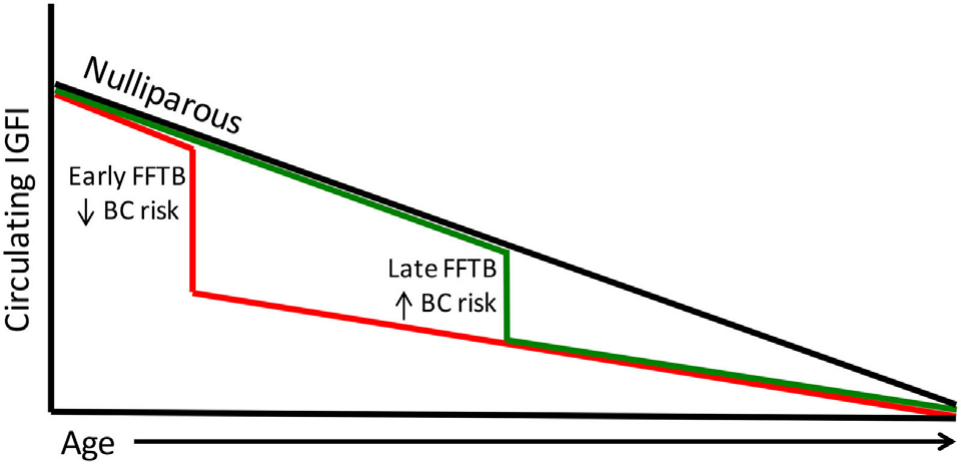
**Circulating IGFI levels parallel breast cancer risk**. Circulating IGFI levels are lower in parous women compared to nulliparous women (black line). A FFTB at an early age (red line) reduces IGFI sooner, foregoing the excess circulating IGFI that women who become pregnant for the first time later encounter (green line). This parallels the data on breast cancer risk, with women who undergo a late FFTB displaying an increased breast cancer risk compared to those who undergo an early FFTB.

The Nurses Health Study data also show an inverse relationship between IGFI levels and age (64). This finding is indicative of the gradual decline of the growth hormone (GH)/IGF axis with age eventually leading to somatopause (65, 66). This is particularly interesting since the highest level of protection against breast cancer occurs in women who encounter their FFTB at an early age, when circulating IGFI levels are highest. Parous women have lower levels of circulating IGFI; therefore, women who undergo parity at a young age would forgo exposure to excessive IGFI levels (Figure 1). Women who undergo their first pregnancy late in life will be exposed to higher circulating IGFI levels until after birth (Figure 1). This parallels the breast cancer risk data in that women who give birth later are also at higher risk for breast cancer.

A link has also been found between IGFI and lobular differentiation. The Nurses Health Studies I and II used 472 women with benign breast disease in a cross-sectional design to show that higher circulating IGFI or a higher IGF:IGFBP3 ratio was associated with decreased odds of having predominantly type 1 lobules (67). Type 1 lobules are the most undifferentiated lobule type as described previously, or possibly represent involuted lobules as speculated by Baer et al. Findings by Russo et al. show that having predominantly type 1 lobules is similar to a nulliparous breast architecture and increases risk for breast cancer (26–28), but other studies show that having predominately type 1 lobules (possibly involuted type 1 lobules) reduces risk for breast cancer (30, 31). While conflicting, these data together suggest a role for IGFI in lobular breast development and cancer risk. It is possible that IGFI contributes breast cancer risk partially by influencing differentiation or involution of breast lobules.

The relationship between IGFI and pregnancy is modified in women afflicted with gestational diabetes and preeclampsia, although the data are conflicting. One study reported an inverse association between IGFI or IGFBP1 and risk for gestational diabetes (68); another reported a positive association between IGFI or IGFBP3 and risk for gestational diabetes (69). While several studies show a lack of a correlation between IGFI and/or IGFBP1 or IGFBP3 and preeclampsia (70–72), the majority report lower IGFI in women with preeclampsia (73–75). Other studies found that higher IGFI is circulating prior to preeclampsia and in gestational hypertension (76, 77). Additionally, data on IGFBPs and preeclampsia vary; Ingec and Giudice report higher IGFBP1 in preeclampsia (70, 73), while Ning and Hietala report lower IGFBP1 in preeclampsia (75, 78). Interestingly, a longitudinal study looking over the course of pregnancy found that compared to healthy controls, during early gestation, IGFBP1 levels were lower in women who later developed preeclampsia, but in late gestation, BP1 levels were higher than controls (79). This observation could be converse for IGFI, in that IGFI could first be higher prior to the clinical condition of preeclampsia [as suggested above (76, 77)] and subsequently reduced with the clinically diagnosed preeclampsia (as the majority of the above studies report).

These findings beg the question whether pregnancy disorders have associations with breast cancer risk. Again, the data are conflicting with many papers finding no association between gestational diabetes or preeclampsia with breast cancer risk (80–84). In some reports, glucose intolerance or gestational diabetes was associated with an increase in breast cancer risk (85, 86), while others found a decrease in risk for breast cancer. One study found that patients with gestational diabetes display lower risk for premenopausal breast cancer (87). In patients with preeclampsia and pregnancy hypertension, a slightly lower breast cancer risk was reported (88), and the second study found substantially lower breast cancer risk in women with severe preeclampsia if they had their first child after the age of 30 (89). Again, the data are conflicting, but there could be multiple important considerations when assessing breast cancer risk, including timing of pregnancy, severity of the disorder during pregnancy, and changes in hormone levels over the course of pregnancy. All of these have yet to be directly tested.

**Figure 2:**
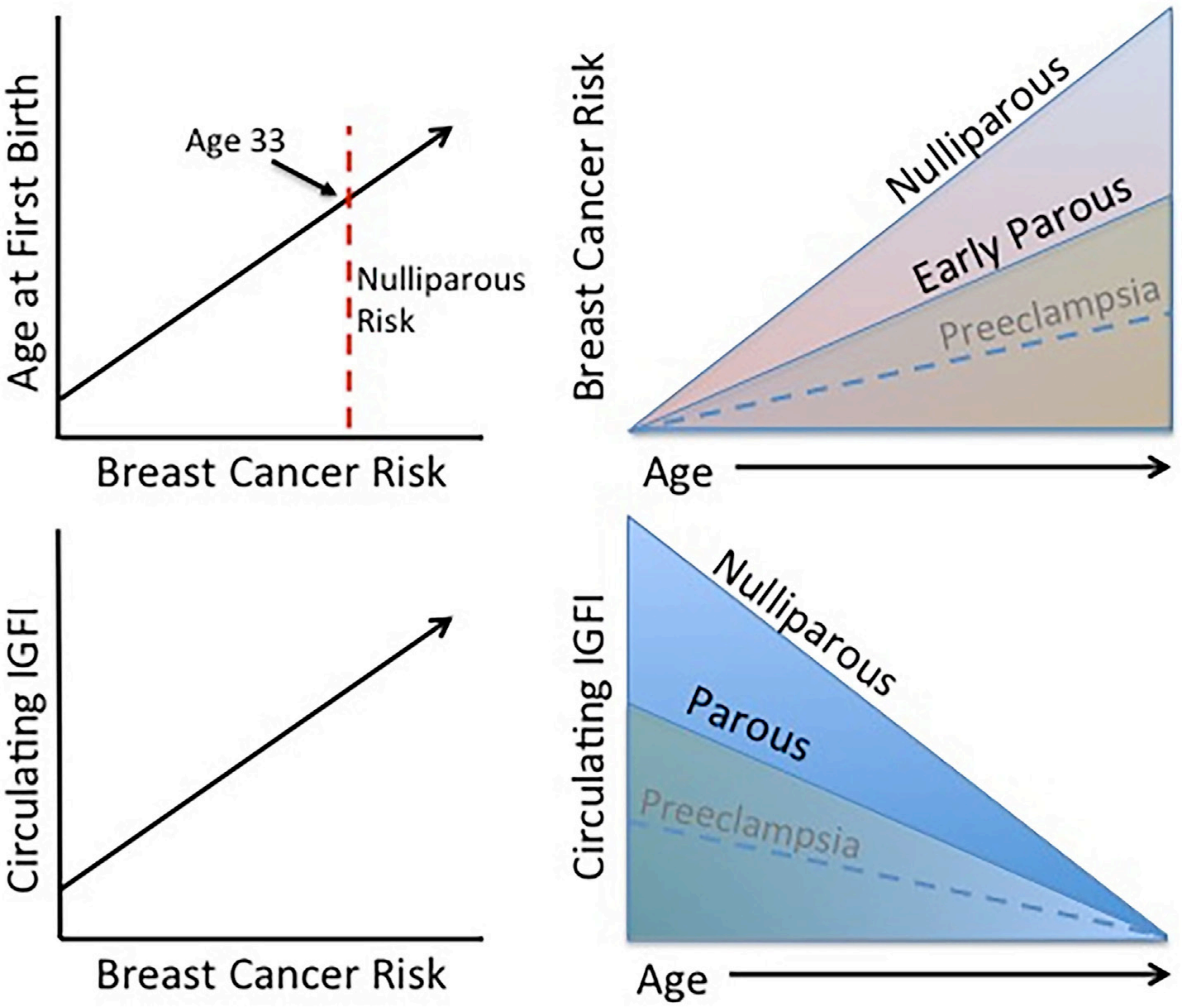
**Established trends associated with the protective effect of pregnancy**. Age at FFTB is positively correlated with breast cancer risk. Women who experience an early parity benefit from a long-term protection against breast cancer compared to nulliparous women. Interestingly, higher levels of circulating IGFI are also positively associated with breast cancer risk and parous women display lower circulating IGFI levels. Although less firmly established, preeclampsia may be associated with a lower breast cancer risk as well as circulating IGFI levels.

In conclusion, long-term exposure to excessive IGFI in circulation leads to an increase in breast cancer risk as described above, and parous women display lower circulating IGFI and breast cancer risk indicating that IGFI contributes to the effect of parity on lifetime breast cancer risk (Figure 2). On the other hand, short-term changes in circulating IGFI during complicated pregnancies (those experiencing preeclampsia or gestational diabetes) may have less of an effect on future risk for breast cancer, as studies investigating IGF levels during these complications are conflicting as are those investigating breast cancer risk. If women who have experienced these complications do in fact have a lower risk for breast cancer than healthy parous controls, it may be through a mechanism unrelated to IGFI or occurring in a specific subset of women.

### Rodent Models

Gene expression studies have shown that growth and proliferation pathways are reduced in parous animals and women. Several studies found *IGF1* expression to be decreased with parity (48, 90–92). In a rodent model, circulating GH was reduced in parous animals leading to a reduction in mammary gland activation of the IGF/GH axis (13, 93). Two rat strains, Sprague Dawley and Wister Furth, were bred at 53 days of age and cannulated at 122 days of age to assess circulating GH. In both strains, average, as well as peak circulating GH, were significantly reduced in parous animals. The reduction in circulating GH translated to reduced activation of GH signaling cascades in the mammary gland, specifically, phosphorylated Jak2, Stat5A, and Akt (93).

Circulating levels of GH as well as IGFI are obligatory for tumor development in certain rodent models. Rats with a genetic deletion of *GH1* (spontaneous dwarf rats, SDR) are completely resistant to carcinogen-induced tumors (94, 95). These animals also display reduced circulating IGFI, and IGFI or GH supplementation rescues carcinogen-induced tumorigenesis (96). In the SDR model, parity or administering E2 and P4 protects against carcinogen-induced GH stimulated tumorigenesis dramatically, reducing tumor incidence from 100% (GH alone) to 16.7% (GH + E2 + P4) and increasing latency from 57 to 138 days, respectively (97). IGFI also abolished the protective effect of pregnancy. Nulliparous and parous animals were treated with IGFI or vehicle for 60 days beginning 7 days prior to carcinogen exposure. Parous animals displayed 16% tumor incidence compared to AMVs displaying 100% tumor incidence. Further, treating parous animals with IGFI resulted in an 83% tumor incidence, thereby eliminating the protective effect of parity (96). IGFI treatment also prevented the induction of lactalbumin in the mammary gland, indicating that full differentiation was not achieved in these animals. These data support the hypothesis that reduced mammary gland differentiation increases tumor susceptibility (96).

## Pregnancy Reprograms the Epigenome, Potentially Contributing to the Protective Effect Against Breast Cancer

Epigenetic changes play a major role in mammary growth and differentiation. DNA is dramatically hypomethylated during lactation to open chromatin and allow expression of milk protein genes (98). A recent study investigating the effect of parity on DNA methylation used MeDIP to pull down methylated regions genome-wide and conducted next-generation sequencing in 19 parous and 16 nulliparous women. They identified FOXA1 to be hypermethylated and silenced with parity. Since FOXA1 is known to colocalize with ER at enhancers, they believe the silencing of FOXA1 will effect ER action, ultimately leading to changes that contribute to the protective effect of parity against breast cancer. This study also found the IGF acid labile subunit (IGFALS), a protein responsible for transport of IGFI in circulation, to be hypomethylated with parity. If this hypomethylation leads to a change in ALS levels in circulation, this could alter the effects of IGFI on target tissues, also contributing to the protective effect of parity.

In our recent study, we identified the *Igf1r* to be hypermethylated and silenced in parous mammary glands. We harvested mammary glands from parous mice and AMVs immediately postpartum (early) and 6 months postpartum (late) (99). This study design enabled us to identify parity-induced differences in DNA methylation, which persist long after pregnancy, therefore possibly contributing to the lifelong protective effect of pregnancy. We utilized a novel targeted hybridization-based approach to identify differentially methylated regions (DMRs). Hybridization probes were designed by Agilent to target regions of the genome likely to be regulated by DNA methylation, covering 3.7 million CpG cites in CpG islands, shores, promoters, enhancers, introns, exons, and intergenic regions. Our analysis identified 624 hypermethylated and 322 hypomethylated genes, which were discovered in the early time point and persisted into the late time point. The *Igf1r* was in the top 10 persistently differentially methylated genes. The DMR in the *Igf1r* was located in intron 2, the largest intron in the gene. This DMR consisted of five CpG sites, all of which displayed increased DNA methylation with parity. *Igf1r* gene expression was significantly reduced at the late time point demonstrating the long-lasting reprograming effect at this locus. We also looked at other IGF pathway members and found several to be significantly hypermethylated at the late time point including the ligands *Igf1, Igfbp4, Irs1, Prlr*, and *Stat5b*. Additionally interesting is that these regions of differential DNA methylation also aligned with regions regulated by histone modifications as evidenced by *in silico* analysis in the UCSC genome browser. When aligning the DMR in the *Igf1r* with several histone marks, peaks in H3K4me1 and H3K4me3 overlap with this portion of intron 2. The same was true for *Irs1, Igf1*, and *Igfbp4*, each aligning with multiple histone marks. These results show that pregnancy induces long-lasting reprograming of the epigenome by altering DNA methylation and possibly histone modifications. These alterations affect the IGF pathway and may contribute to the protective effect of parity.

As previously mentioned, histone modifications are also likely to play a role in reprograming the breast epigenome during pregnancy. In the breasts of nulliparous women, nuclei were large and euchromatic in contrast to parous women displaying small heterochromatic nuclei with strong methylation of histones at repressive marks H3K9me2 and H3K27me3 (100). One study identified H3K27me3 and its writer EZH2 to be increased during pregnancy and to decline during late pregnancy, particularly in the mammary stem cell population. This regulation was tightly correlated with gene expression of pregnancy-associated targets such as *Csn2* and *Wap* and was mediated by P4 (101).

## Conclusion and Future Directions

**Figure 3:**
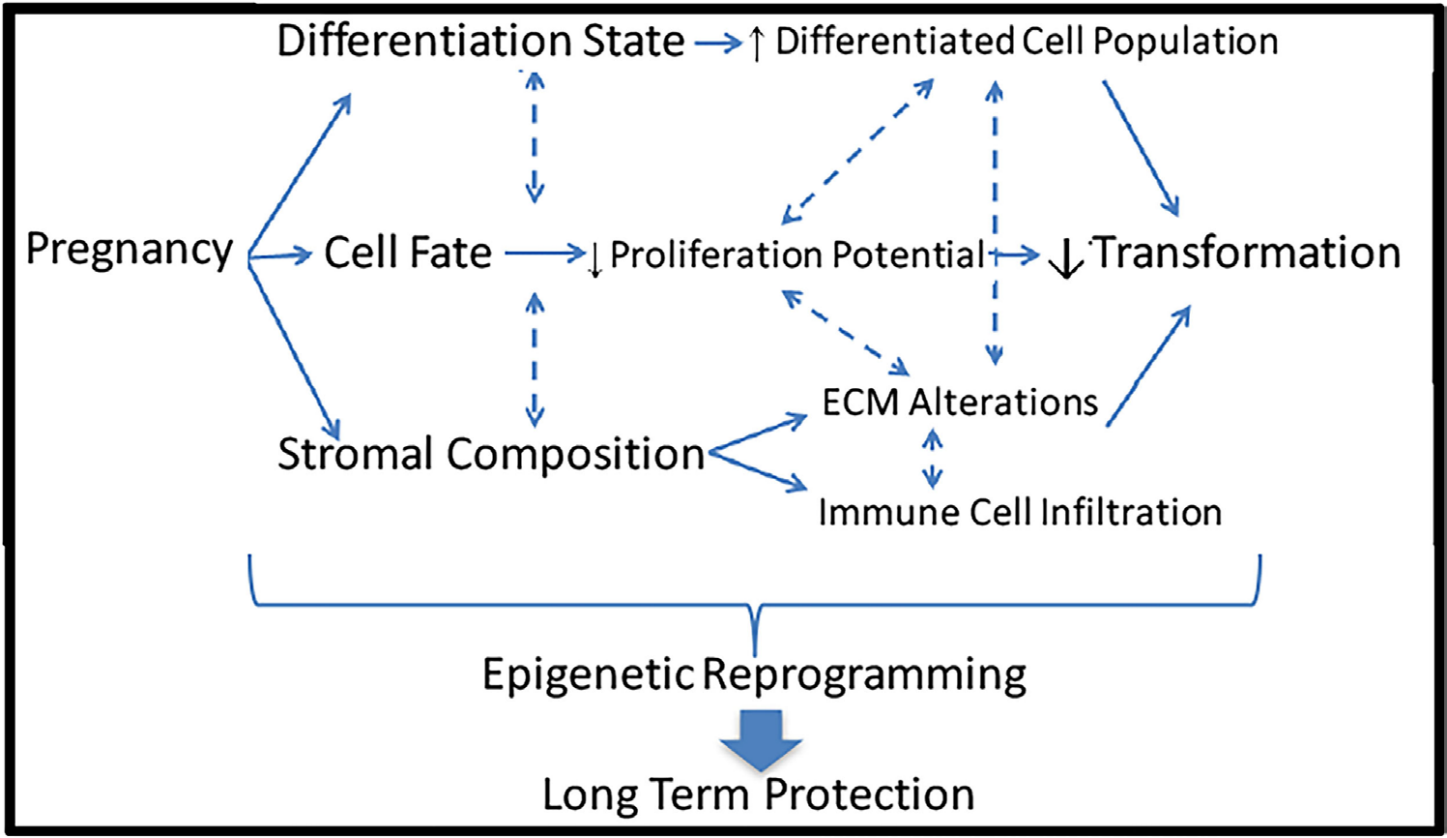
**Potential mechanisms underlying the protective effect of pregnancy against breast cancer**. Pregnancy induces a multitude of dramatic changes in the breast including differentiation state, cell fate, and stromal composition. Each of these alterations could lead to reduced risk of cellular transformation and breast cancer induction. All of these mechanisms could be controlled by epigenetic reprograming, leading to long-term protective effects.

Investigations probing the mechanism underlying the protective effect of pregnancy against breast cancer have been conducted in many areas including lobular differentiation, cell fate, and stromal composition. However, no single cause has been identified, and likely these mechanisms complexly interact to elicit the protective effect. The long-lasting effect of an early FFTB on reducing breast cancer risk implies that a relatively persistent modification may contribute to this phenomenon such as epigenetic reprograming (Figure 3).

While an early FFTB provides the greatest protection against breast cancer compared to any other risk factor, a method to exploit this phenomenon for a preventative strategy has not been developed. Currently, the most widely used preventative measure is tamoxifen, but due to toxicities, this is only available to high-risk women. Tamoxifen therapy causes unpleasant side effects leading to a drastic diminishment in its use in more recent years (102, 103). There are investigations into IGF1R inhibitors as preventative therapies, but these are toxic as well and therefore again only will be helpful in high-risk women (104–106). With the age of FFTB rising and the lack of preventative options, discovering the most important mechanisms underlying the protective effect of an early FFTB is imperative to developing prevention strategies for young healthy women (11, 107). The most effective way to eradicate a disease is through prevention; therefore, new preventative avenues for young healthy women are essential to reducing breast cancer incidence.

## Author Contributions

TK conducted literature searches and wrote the review article.

## Conflict of Interest Statement

The author declares that the research was conducted in the absence of any commercial or financial relationships that could be construed as a potential conflict of interest.
